# Effectiveness of a web-based intervention for injured claimants: a randomized controlled trial

**DOI:** 10.1186/1745-6215-14-227

**Published:** 2013-07-20

**Authors:** Nieke A Elbers, Arno J Akkermans, Pim Cuijpers, David J Bruinvels

**Affiliations:** 1 Department of Law, VU University, De Boelelaan 1105, 1081 Amsterdam, HV, The Netherlands; 2 Department of Clinical Psychology, VU University, De Boelelaan 1105, 1081 Amsterdam, HV, The Netherlands; 3 EMGO Institute, VU University Medical Center, Van der Boechorststraat 7, 1081 Amsterdam, BT, The Netherlands; 4 Netherlands Society of Occupational Medicine (NVAB), Churchilllaan 11, 3527 Utrecht, GV, The Netherlands

**Keywords:** Injury compensation, Web-based intervention, Randomized controlled trial, E-health, Empowerment

## Abstract

**Background:**

There is considerable evidence showing that injured people who are involved in a compensation process show poorer physical and mental recovery than those with similar injuries who are not involved in a compensation process. One explanation for this reduced recovery is that the legal process and the associated retraumatization are very stressful for the claimant. The aim of this study was to empower injured claimants in order to facilitate recovery.

**Methods:**

Participants were recruited by three Dutch claims settlement offices. The participants had all been injured in a traffic crash and were involved in a compensation process. The study design was a randomized controlled trial. An intervention website was developed with (1) information about the compensation process, and (2) an evidence-based, therapist-assisted problem-solving course. The control website contained a few links to already existing websites. Outcome measures were empowerment, self-efficacy, health status (including depression, anxiety, and somatic symptoms), perceived fairness, ability to work, claims knowledge and extent of burden. The outcomes were self-reported through online questionnaires and were measured four times: at baseline, and at 3, 6, and 12 months.

**Results:**

In total, 176 participants completed the baseline questionnaire after which they were randomized into either the intervention group (n = 88) or the control group (n = 88). During the study, 35 participants (20%) dropped out. The intervention website was used by 55 participants (63%). The health outcomes of the intervention group were no different to those of the control group. However, the intervention group considered the received compensation to be fairer (*P* <0.01). The subgroup analysis of intervention users versus nonusers did not reveal significant results. The intervention website was evaluated positively.

**Conclusions:**

Although the web-based intervention was not used enough to improve the health of injured claimants in compensation processes, it increased the perceived fairness of the compensation amount.

**Trial registration:**

Netherlands Trial Register NTR2360

## Background

There is considerable evidence showing that injured people involved in a compensation process show less physical and mental health than people with similar injuries who are not involved in a compensation process [1,2]. One explanation for this reduced recovery is that the legal procedure surrounding the award of compensation is very stressful for the claimant [3]. There are signs that claimants feel hampered by a lack of information and lack of communication [4] and that they are burdened by the many medico-legal assessments in which they have to prove their injury [5]. Furthermore, they experience stress from the attitude of lawyers and insurance companies [6]. Reduced recovery because of the compensation process is a problem, of course, for the injured people themselves, but also for society as it implies higher health-care costs and increased income replacement benefits. It is obvious that this problem needs to be addressed. This study aims to contribute to improvements.

At the time we started developing the intervention, there was no intervention study that addressed compensation stress. Recently, during our study, two interventions have been published. One was about an Australian motor vehicle insurance company that implemented a new claims settlement procedure, that is, effective communication, early intervention, screening for adverse factors, and a focus on early return to work. This new approach achieved a reduction in depression and improved return to usual activities as compared to claim handling as usual [7]. The second study was a pilot conducted by Dutch insurance companies that tested a new claims settlement approach for whiplash injury claims. For one year, all legal and medical discussions were postponed, claimants were supported by case managers, and costs were fully compensated by the participating insurance companies. This approach increased client satisfaction as compared to care as usual [8]. These two studies show that a change to claims settlement can improve claimants’ health and satisfaction. However, this requires substantial effort and an extensive culture change. Instead, we wanted to develop an intervention that was easily accessible for claimants. We were guided by the fact that claimants indicated that they were negatively affected by a lack of information, lack of communication and lack of involvement in the compensation process.

The type of intervention we considered to meet our goals was an e-health intervention. E-health interventions have been developed for both physical and mental health problems - both of which are applicable to our study population. In general, these interventions mostly contain information and cognitive behavioral therapy elements [9]. They have been found to be effective in reducing pain, depression and anxiety [10,11] and they are able to increase self-efficacy, knowledge and communication skills [12]. Although e-health interventions involve some problematic issues such as high dropout by participants, lack of regular website access, and a need for some interaction in order to be effective [13], they are as effective as face-to-face treatments [14,15]. They even have several advantages over face-to-face interventions: they are anonymous, the costs are low, and they can be accessed at any time and any place [16]. Furthermore, they are very suitable for mild symptoms [17], which is probably the case in the current study population. Our e-health intervention contained (1) information about the claims settlement process, and (2) a problem-solving therapy about how to cope with problems, such as those arising in the compensation process. It was expected that this intervention would improve feelings of control (empowerment), self-efficacy, health status, perceived justice, knowledge, and the ability to work.

## Methods

### Participants

Participants were individuals aged over 18 at the time of enrolment, who had been injured in a traffic crash less than two years ago and were claiming compensation for financial losses. Furthermore, they were required to speak Dutch and to have access to the internet. Participants were recruited via three Dutch personal injury claims settlement offices located in Alphen aan den Rijn, Amsterdam and Amersfoort.

In the Netherlands, car crash compensation claims are settled on the basis of classical tort law. Claimants are required to prove that somebody else was liable for the accident and that there is a causal relationship between accident, injury and damages. After liability and causality are established, the wrongdoer’s insurance company pays for (additional) loss of income (to a certain level, employees receive social security benefits), travel and household support services, additional medical services (to a certain level, claimants’ health insurance pays for health services), rehabilitation and disability services, legal fees, and pain and suffering. Damages are paid in a lump sum, but claimants normally receive some advance payments. As in most countries, the majority of claims (95%) are settled out of court.

A power calculation showed that 170 (2 × 85) participants would be sufficient to detect a medium effect size of empowerment between two groups, using a power of 80% and an alpha of 5%, and taking into account a loss to follow-up of 25%.

### Procedure

The claims settlement offices were asked to send their clients an information leaflet by email or by post. Clients applied for the study by completing an online registration form and providing informed consent on the website http://www.gripopmijnzaak.nl (‘claim under control’), hosted by the VU University. After they completed the online registration form and confirmed informed consent, the inclusion criteria were checked. Eligible participants were sent the baseline questionnaire. Participants who completed this questionnaire were randomized into either the intervention or the control condition.

The randomization scheme was created by a computerized random block generator, creating fixed blocks of 20. Two randomization schemes were created: one for participants whose injury occurred less than 1 year ago and one for those whose injury occurred 1 to 2 years ago. This stratified randomization ensured that the length of time since injury was equally divided over the intervention and control conditions. Randomization was performed by the principle investigator.

Participants received the login codes for either the intervention or the control website. Neither participants nor their lawyers were told which group they were in, so they were considered to be blind for group assignment. In total, there were four online questionnaires: at baseline, after 3 months, after 6 months and after 12 months. Twelve months is the average duration of compensation processes. Participants received a 20 euro voucher if they completed all four questionnaires. About halfway through the study, all participants received an online information leaflet in order to increase website usage. The study protocol has been published previously [18]. The Medical Ethics Committee of the VU University medical centre approved the study protocol.

### Intervention and control website

The intervention website consisted of three modules: (1) information about the compensation process (49 pages), (2) a five-lesson problem-solving therapy, and (3) 10 frequently asked questions with answers (one page). The information module contained an overview of the four phases of the compensation process, including the important definitions, steps, length of time, and bottlenecks. The other information topics concerned what to expect from lawyers, what to expect from insurance companies, the different social security regulations, and what the options are in the case of conflict [18].

The problem-solving therapy consisted of five lessons, which explained to participants how to make a step-by-step plan to solve problems, how to communicate efficiently, how to recognize thinking errors, and how to cope with unsolvable problems [19,20]. Each lesson contained examples of other claimants’ problems and their solutions. Examples of problems were: having to cope with (permanent) injury, being traumatized by the crash, or being subjected to frequent medical assessments. Other examples were: being burdened by financial problems because the insurance company is behind in paying advances, or being accused of contributory negligence. Each lesson also included some assignments in which participants could tackle their own problems. Participants who completed these assignments were given feedback via email by the principal investigator [18].

The website was evaluated in a focus group with lawyers and insurance companies, both of whom voiced their expectation that the website would meet the claimant’s needs. The intervention was also pilot tested by eight claimants, who rated the website positively. They all indicated that they would use the information module, and three out of eight would use the e-coach [18].

The control website contained links to already existing information and support websites only (eight pages in total). For example, we included links to the website of the Dutch Personal Injuries Board, the Wikipedia page about personal injury and the social security website. We also referred participants to the website of the Dutch victim support organization and the whiplash association’s website. The control condition can be regarded as care as usual, because lawyers refer to these websites and they can easily be found when googling. Both the intervention website and the control website were accessed on http://www.gripopmijnzaak.nl. After the login page, the intervention group was assigned to the intervention content and the control group to the control website. The content of both websites was frozen.

### Outcome

The primary outcome measures were empowerment, measured by the mastery scale (α = 0.68) [21], and self-efficacy about the accident, the injury and the compensation process, which was assessed by a self-developed questionnaire (α = 0.92) [22]. Health status was assessed by the EuroQol (α = 0.64) [23], and by the depression, anxiety, and somatic symptoms subscale of the symptom checklist SCL-90 [24]. Procedural, interactional, informational and (if the claim was settled) distributive justice were determined by the organizational justice scale [25]. These scales investigated the perceived fairness of the compensation procedure (α = 0.88), the interaction with lawyers (α = 0.83) and insurance companies (α = 0.92), the information provided (α = 0.96), and (if the claim was settled) the compensation received (α = 0.94).

Ability to work was measured by the first three items of the Work Ability Index, assessing current ability (including studies, volunteer work and housekeeping) compared to highest ability ever, and work ability in relation to physical and mental demands [26]. Also examined was whether claimants knew about what was going on during the claims settlement process (‘claim knowledge’) (α = 0.89) and whether they perceived the compensation process to be a burden. When they indicated that their claim was settled or when they received the final questionnaire, the participants were asked to grade the website and to indicate the amount of compensation they received or expected. Furthermore, the participants’ lawyers were asked to rate the communication with that client [18].

Ten questions were added to the final questionnaire in order to enable evaluation of the intervention website. The first five questions were about the website as a whole, discussing its appearance, the language used, the usefulness of the information, and the structure. The last five questions concerned the e-coach module: whether it was user-friendly, whether the method was appealing, whether it took too much time, whether the e-coach was needed, and whether the computer is a suitable medium for dealing with worries and problems. The answer scale ranged from 1 to 10 (1 = totally disagree, 10 = totally agree). These questions were put to the intervention group whose claim was still pending.

### Statistical analysis

Attrition was defined as not completing the follow-up questionnaires. Website usage was defined as having logged in to the website. Short-term (that is 3 months after baseline) and long-term (that is 12 months after baseline) differences between the intervention and control groups were analyzed using linear multivariate regression analyses. Baseline corrections were applied. The analyses were conducted according to the intention-to-treat principle. Missing data were imputed using the last value carried forward method. Additionally, Generalized Estimation Equation (GEE) analyses were performed on the non-imputed dataset [27] to investigate the overall effect of the intervention on all outcome measures.

To examine the effect of the intervention on the distributive justice scale, which was only completed if the participants indicated that their claim was settled, an independent *t* test was performed on the settled claims. An independent *t* test was also used to compare the evaluation grade of the intervention and the control website, and to investigate whether there was a difference regarding the communication grade that was given by the participants’ lawyers. Finally, a subgroup analysis was conducted, comparing the outcomes of the intervention users versus intervention nonusers by means of linear regression and GEE analysis. Data was analyzed using SPSS version 18.0.3 (IBM SPSS Statistics, Chicago, IL, USA). To correct for the multiple analyses, *P* < 0.01 was used.

## Results

### Participants

**Figure 1:**
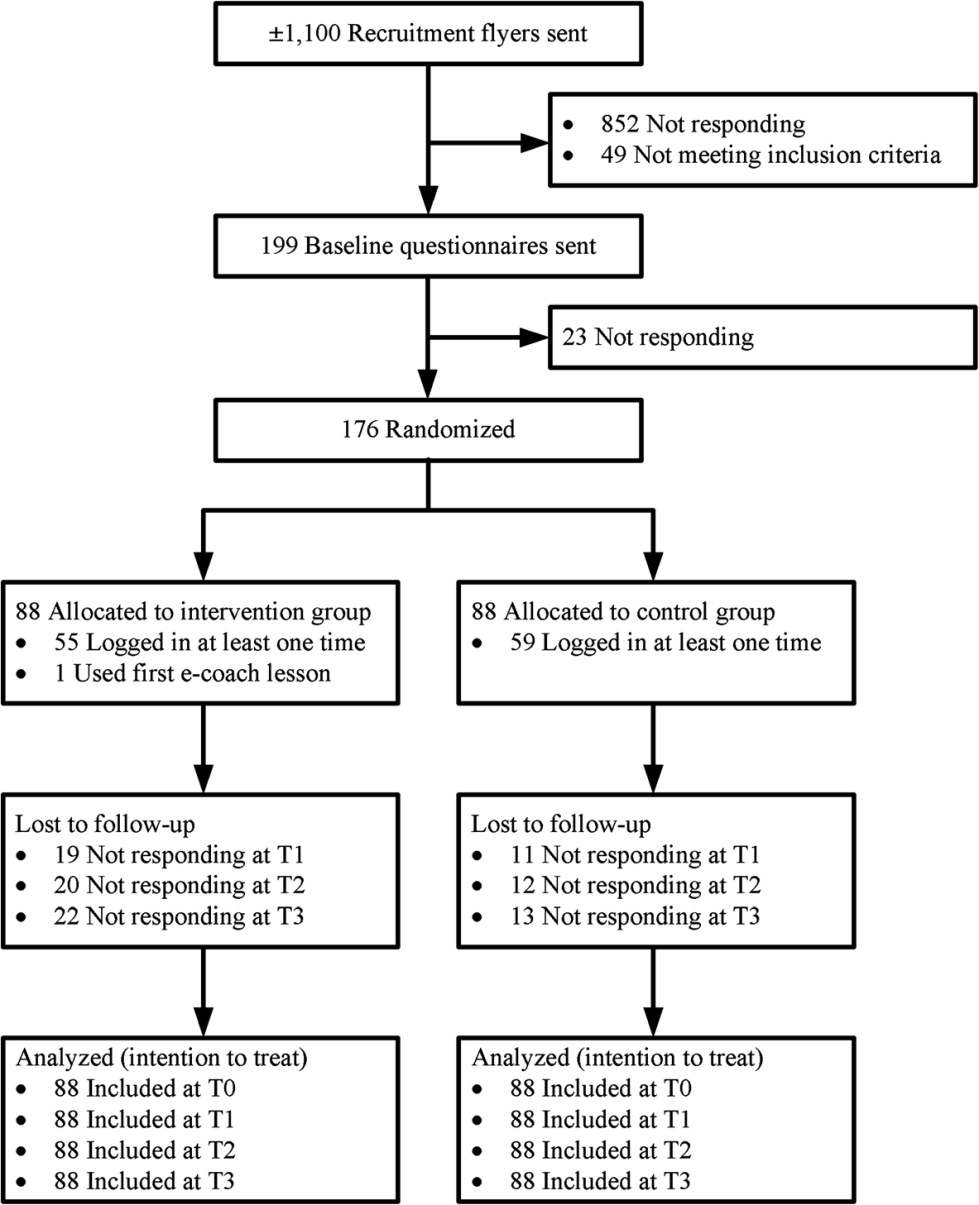
Participant flow chart.

Recruitment took place from October 2010 until March 2011. About 1,100 clients were sent the recruitment flyer. In total, 248 people indicated interest in enrolment in the study by completing the online registration form. Of these, 49 were excluded because they did not meet the inclusion criteria. The remaining 199 respondents were sent the baseline questionnaire. Of these, 23 were excluded because they did not complete the baseline questionnaire. The remaining 176 participants were included in the study and subsequently randomized to either the intervention (n = 88) or the control group (n = 88). The participant flow is displayed in Figure 1.

The mean age of the participants was 48.7 years and 53% were male. Time since injury was 12 months on average. Time involved in the compensation process was 10 months. Forty-two percent of participants were hospitalized, with an average hospital stay of 9.3 days. Thirty-two percent of the participants had whiplash injury. An overview of participant characteristics is provided in Table 1. There were no significant differences between the intervention and control group characteristics; this implies that randomization was successful.

**Table 1:** Participant characteristics

		**All (n = 176)**	**C (n = 88)**	**I (n = 88)**	***P***
		**M (SD) or%**	**M (SD) or%**	**M (SD) or%**	
Age		48.6 (14.7)	48.3 (14.5)	48.9 (15.0)	0.77
Gender	Male	53.4	56.8	50.0	0.37
Country of birth	The Netherlands	96.0	95.5	96.6	0.70
Work	Employer	65.3	72.7	58.0	0.10
	Self-employed	13.1	9.1	17.0	
	Unemployed	21.6	18.2	25.0	
Education	Lower	22.2	22.3	23.0	0.81
	Middle	55.1	56.5	41.4	
	Higher	22.7	21.2	35.6	
Time since injury		11.9 (7.2)	12.0 (7.4)	11.8 (7.2)	0.89
Traffic participant	Motorized	71.0	70.5	71.6	0.87
Blaming offender	Not at all – a little	12.0	15.3	9.2	0.67
	Neutral	7.4	5.9	8.0	
	Quite – very much	80.7	78.8	82.7	
Injured body part	Shoulder, arm, hand	50.6	53.4	47.7	0.45
	Head or neck	50.0	48.9	51.1	0.76
	Hip, leg, foot	49.4	53.4	45.5	0.29
	Trunk or back	30.1	25.0	35.2	0.14
Hospitalization		42.0	45.5	38.6	0.36
Number of days in hospital		9.3 (11.0)	8.0 (9.3)	10.9 (12.6)	0.26
Whiplash injury		31.8	28.4	35.2	0.33
Claims settlement	Korevaar Van Dijk	44.9	46.6	43.2	0.83
office	Hofmans	46.0	45.5	46.6	
	Kloppenburg	9.1	8.0	10.2	

*C*, control group; *I*, intervention group; *M*, mean; *SD*, standard deviation. The *P* value indicates whether the participant characteristics differ between groups.

### Attrition

Attrition rates were 17% (n = 30) at 3 months after baseline, 18% (n = 32) at 6 months after baseline, and 20% (n = 35) at 12 months after baseline. The attrition was not significantly different in the intervention group compared to the control group (after 3 months: χ^2^ = 2.57, *P* = 0.11; after 6 months: χ^2^ = 2.44, *P* = 0.12; after 12 months: χ^2^ = 2.89, *P* = 0.09). Participants who dropped out of the study were no different from those who remained regarding baseline outcome measurements, communication grade or website evaluation.

In total, 72 participants (41% of the sample) indicated that their claim was settled during the study. Whether dropout was associated with settlement could not be investigated, because participants who dropped out were scored as such because they did not fill in the follow-up questionnaires. However, 69 of the 72 participants who indicated that their claim was settled did complete the questionnaires, so there does not seem to be an association between settlement and dropout.

### Effect of the intervention

**Figure 2:**
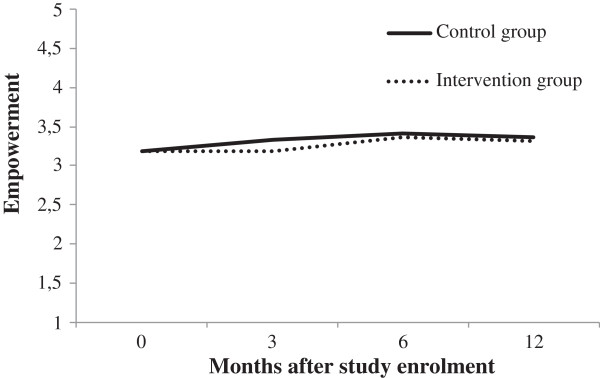
Empowerment outcomes over time for the intervention and control group.

The linear regression analyses examining short-term (3 months) and long-term (12 months) effects of the intervention showed that the intervention group did not score better than the control group on most of the outcome measures, that is self-efficacy, procedural justice, health status, depression, anxiety, somatic symptoms, ability to work, or extent of perceived burden (see Table 2). There was a trend (*P* > 0.01) showing that the intervention may have a short-term negative effect on empowerment (β = −0.12, *P* = 0.03) and on claim knowledge (β = −0.14, *P* = 0.02) but the effect sizes were small and the trend was no longer present after 12 months. The GEE analyses did not reveal significant differences. To illustrate the course of one of the outcome measures, Figure 2 shows the (non-imputed) empowerment score over time.

**Table 2:** Linear regression analyses investigating short- and long-term effects of the intervention

**Outcome measure [range]**	**C/I**	**Baseline**	**3 months**	**6 months**	**12 months**	**Short-term effect**		**Long-term effect**	
						**(3 months)**		**(12 months)**	
		**M (SD)**	**M (SD)**	**M (SD)**	**M (SD)**	β	***P***	β	***P***
Empowerment [1-5]	C	3.19 (0.63)	3.31 (0.67)	3.40 (0.57)	3.37 (0.56)	-.012	0.03	−0.10	0.10
	I	3.19 (0.71)	3.15 (0.71)	3.27 (0.71)	3.24 (0.74)				
Self-efficacy [0–10]	C	7.48 (2.21)	7.68 (1.86)	7.82 (1.92)	7.80 (1.89)	−0.02	0.64	−0.06	0.27
	I	7.49 (2.10)	7. 59 (2.40)	7.57 (2.37)	7.54 (2.32)				
Procedural justice [1-5]	C	3.60 (0.93)	3.45 (0.95)	3.47 (1.01)	3.49 (0.88)	0.01	0.99	−0.02	0.70
	I	3.54 (1.05)	3.41 (1.12)	3.38 (1.07)	3.41 (1.01)				
Interactional justice [1-5]^a^	C	4.70 (0.60)	4.62 (0.72)	4.70 (0.55)	4.68 (0.55)	−0.05	0.43	−0.05	0.43
	I	4.75 (0.57)	4.57 (0.77)	4.67 (0.62)	4.64 (0.68)				
Informational justice [1-5]	C	4.27 (0.86)	4.14 (0.93)	4.13 (0.92)	4.10 (0.85)	−0.06	0.27	−0.05	0.41
	I	4.42 (0.87)	4.14 (1.03)	4.14 (0.99)	4.13 (1.03)				
Interactional justice [1-5]^b^	C	3.34 (1.20)	3.38 (1.33)	3.42 (1.28)	3.34 (1.29)	0.02	0.72	0.08	0.16
	I	3.19 (1.12)	3.30 (1.33)	3.33 (1.36)	3.40 (1.30)				
Burden [1-10]	C	5.89 (2.79)	5.88 (2.60)	5.57 (2.64)	5.82 (2.61)	−0.05	0.40	−0.01	0.87
	I	5.52 (2.56)	5.39 (2.75)	6.65 (2.83)	5.57 (2.92)				
Depression [1-5]	C	1.65 (0.80)	1.67 (0.77)	1.56 (0.68)	1.61 (0.75)	−0.01	0.81	0.02	0.68
	I	1.72 (0.86)	1.73 (0.88)	1.69 (0.82)	1.69 (0.82)				
Anxiety [1-5]	C	1.52 (0.70)	1.51 (0.63)	1.18 (0.66)	1.47 (0.68)	0.03	0.37	0.02	0.56
	I	1.60 (0.81)	1.64 (0.87)	1.58 (0.79)	1.58 (0.76)				
Somatic complaints [1-5]	C	1.79 (0.65)	1.75 (0.66)	1.67 (0.64)	1.66 (0.67)	0.03	0.40	0.06	0.21
	I	1.84 (0.75)	1.84 (0.76)	1.80 (0.75)	1.78 (0.73)				
EuroQol VAS [0–10]	C	6.44 (1.93)	6.66 (1.89)	6.84 (2.07)	6.92 (1.91)	−0.05	0.35	−0.06	0.31
	I	6.11 (2.10)	6.22 (2.14)	6.36 (2.17)	6.45 (2.28)				
Work ability VAS [1-10]	C	6.17 (2.36)	6.39 (2.13)	6.67 (2.16)	6.61 (2.17)	−0.08	0.10	−0.02	0.71
	I	5.68 (2.41)	5.64 (2.57)	5.90 (2.63)	6.17 (2.46)				
Claim knowledge [1-5]	C	3.01 (0.93)	3.27 (0.96)	3.26 (1.05)	3.30 (1.01)	−0.14	0.02	−0.10	0.11
	I	3.08 (0.95)	3.05 (0.95)	3.03 (1.04)	3.13 (1.06)					

^a^Regarding the lawyer; ^b^regarding the insurance company. *C*, control group; *I*, intervention group; *M*, mean; *SD*, standard deviation; *VAS*, visual analogue scale. M (SD) are raw scores, but for the analyses, data are imputed and corrected for baseline differences. Significance level was set at *P* < 0.01.

The independent *t* tests analyzing the distributive justice scale showed that the intervention group experienced more distributive justice than the control group, *t* (58) = −2.82, *P* < 0.01. The *t* test represented a medium effect size (r = 0.35). The intervention group did not receive (*P* = 0.40) or expect (*P* = 0.79) a significantly different compensation amount than the control group. Finally, the lawyers did not grade the communication with the intervention group better than the communication with the control group (*P* = 0.27). Means, standard deviations, and *t* tests are displayed in Table 3.

**Table 3:** **Independent *t *tests investigating distributive justice, the received/expected compensation amount, and communication graded by the lawyer**

**Outcome measure [range]**	**C/I**	**M (SD)**	***t *test**
Distributive justice [1-5]	C	3.26 (1.25)	t (58) = −2.82, *P* <0.01
	I	4.00 (0.79)	
Compensation amount received	C	9,448 (18,042) Euro	t (51) = 0.85, *P* = 0.40
	I	5,893 (9,302) Euro	
Compensation amount expected	C	36,652 (85,502) Euro	t (47) = −0.27, *P* = 0.79
	I	45,557 (134,713) Euro	
Communication grade [1-10]	C	7.4 (1.1)	t (159) = −1.11, *P* = 0.27
	I	7.6 (1.1)	

*C*, control group; *I*, intervention group; *M*, mean; *SD*, standard deviation. These outcomes were measured either at 12 months after baseline or after the participants indicated that their claim was settled.

Short-term and long-term linear regression subgroup analyses in which the intervention users (n = 55) were compared to the intervention nonusers (n = 33) showed that the intervention users did not score different from intervention nonusers on any of the outcome measures. The GEE subgroup analyses did not show any differences either.

### Website usage

Of all 176 participants, 114 people logged in to the website (65%). Those who logged in, tended more often to be female (χ^2^ = 4.75, *P* = 0.03). The majority (55%) of people who logged in, entered the website only once or twice, and most of them did so within two weeks after receiving the login code. Website usage was associated with whether the claim was pending or settled, because claimants whose compensation claim was pending were more inclined to spend time on the website (mean (M) = 5.70 minutes, standard deviation (SD) = 13.52) than those whose claim was settled (M = 2.51, SD = 4.23), *t* (108) = 2.05, *P* = 0.04.

Intervention website users spent on average 8.7 minutes on the website; the control group, 4.1 minutes. Both groups viewed about 10 web pages. The information about the compensation process phases was read by 55 people in the intervention group. The pages about what to expect from their lawyer or from the insurance company were viewed by 19 participants: social security information was read by 12, and 16 participants were interested in the information about conflict solutions. In total, 39 participants in the intervention group clicked on the e-coach tab, but only one actually started the e-coach course, completing only the first lesson after completing the final questionnaire. The frequently asked question tab was accessed by 41 people in the intervention group.

### Website evaluation

The intervention group graded the website better (M = 7.5) than the control group (M = 6.9), *t* (104) = −2.76, *P* < 0.01. The appearance, language, usefulness, and structure of the intervention website were rated fair to good, that is, the averages ranged between 7.3 and 8.1 on a 1 to 10 scale. The amount of information was graded fair, that is, 6.1 on a scale in which 1 was lowest and 10 was highest.

The e-coach method, which was described on the first page of the e-coach file tab, which was clicked on by 75% of the people who logged onto the website, was considered to be fairly user-friendly (M = 7.6), quite appealing (M = 7.2), and the computer was considered to be a reasonably suitable instrument for dealing with worries and problems (M = 6.7). However, the e-coach course also takes up quite some time (M = 6.2) and some participants indicated that they did not need the e-coach (M = 7.3).

## Discussion

This study investigated whether a web-based intervention could empower injured claimants suffering from distress caused by the compensation procedure. It was found that the intervention group considered the received compensation amount to be fairer than the control group (*P* < 0.01). This does not seem to be due to the size of the compensation amount, because the intervention group received a statistically similar compensation amount to the control group. Therefore, it seems reasonable to conclude that the intervention website provided a better picture about what compensation amount is fair. However, this finding should be interpreted with caution because the number of participants in the distributive justice analysis was relatively small (n = 60). Remarkably, participants whose claims were pending expected a much higher compensation amount than what was actually received in the settled claims, which may imply that the overall expectation regarding the size of the compensation amounts may not be realistic. However, that does not alter the fact that the intervention website apparently increased the perceived fairness of the received amount.

In contrast to what was hypothesized, the intervention did not have a significantly positive effect on any of the other outcomes. Three months after baseline, the intervention even had a negative effect on empowerment (*P* = 0.03) and claim knowledge (*P* = 0.02), although the latter may be interpreted in an exploratory manner. However, the effect sizes were small and there was no negative effect in the long term (that is at 12 months), so in general we conclude that there is no intervention effect.

The first plausible explanation for a lack of intervention effect is low website usage. About 35% of the intervention group did not log in to the website. Those who did log in mostly did so only once or twice. Only one participant completed one e-coach lesson. Low website usage could be explained by the fact that the lawyers could not refer their clients to information or support on the website, which could not be done because we wanted to conduct a blind randomized controlled trial. Low website usage could also have been caused by the fact that the participants in the sample were somewhat older than average (48 versus 39): generally, older people are not as familiar with the internet as younger people. Low website usage did not seem to be caused by any dislike of the appeal, content, or structure of the website, because the questions evaluating these aspects were answered quite positively, except that they indicated not needing an e-coach (7.3 on a scale from 1 to 10) (which is remarkable because their health was significantly poorer than that of the average Dutch population (6.3 versus 8.3 [28]).

A second explanation why this study did not show an intervention effect could be that the (legal) professionals involved did not respond well to the empowered claimants, as was found in another study [29]. However, we have not asked participants whether this was the case. A final explanation may be that participants consciously or unconsciously did not want to get better as long as the claims settlement lasted (secondary gain [30]). However, previous studies have shown that claimants seeking compensation have similar treatment participation and treatment outcomes to their non-compensation-seeking counterparts [31,32].

An important strength of this study is that the trial setup was double blinded, which is quite unique in e-health studies [33]. Another strength is the randomized controlled trial (RCT) design, because RCTs had been nonexistent in compensation studies [34,35]. Other positive features of this study are the high number of participants, an acceptable (20%) loss to follow-up and good registration of website usage. An important limitation, however, was a possible selection bias: it could be that only very satisfied claimants responded. Future studies should conduct a feasible nonresponse investigation. Secondly, the sample was somewhat older than average and the response rate was quite low (16%), which may limit the generalizability of study results to the general claimant population. Finally, compensation schemes differ between countries [36], so the current results may not be applicable to other compensation schemes (although most compensation schemes for traffic accidents are based on tort, and in most countries the majority of claims are settled out of court [37]).

Although our e-health intervention did not succeed as such, we would still like to encourage clinical psychologists to ask those clients who are involved in a compensation process whether they are burdened by any aspect of the compensation claim. We believe ‘compensation stress’ does not get enough attention in current therapies and some claimants could use some coping and problem-solving strategies. Legal professionals may learn from this study that providing adequate information about the compensation process and the possible damages that claimants are entitled to, may increase the claimants’ perceived fairness about the compensation amount that they receive. Finally, a lesson from this study for (e-health) researchers is that once again e-health research has been shown to have not yet overcome one of its major problems, that is, lack of usage. Maybe this particular population is not ready for online coaching. However, it is a fact that improving the claimants’ health is needed, so it is important to investigate whether e-health interventions can achieve that in another study design, for example, by conducting an effectiveness study involving people who actually ask for help.

## Conclusions

In contrast to what was hypothesized, the intervention did not have any positive effect on claimants’ health. The low (e-coach) website usage is likely to be the reason for this lack of effect. On the other hand, the intervention group perceived their compensation amount to be fairer, so it seems that the information module was somewhat beneficial. As the costs of the website are low, and maintenance is not labor-intensive, the information on the website could still be made generally accessible to injured people who are involved in compensation procedures. The value of the e-coach module should be investigated in a different study design and/or with a sample of participants who actually require help.

## Competing interests

The authors declare they have no competing interests.

## Authors’ contributions

NE was responsible for the design, recruitment, data analysis, interpretation of data and drafting the article. AA, PC and DB contributed to the development of the study design and interpretation of data and commented on the manuscript. All authors read and approved the final version of the manuscript.
